# The laminar integration of sensory inputs with feedback signals in human cortex

**DOI:** 10.1016/j.bandc.2016.06.007

**Published:** 2017-03

**Authors:** Lucy S. Petro, Lars Muckli

**Affiliations:** Centre for Cognitive Neuroimaging, Institute of Neuroscience and Psychology, University of Glasgow, 58 Hillhead Street, Glasgow G12 8QB, Scotland, United Kingdom

**Keywords:** Human functional brain imaging, Cortical feedback

## Abstract

The cortex constitutes the largest area of the human brain. Yet we have only a basic understanding of how the cortex performs one vital function: the integration of sensory signals (carried by feedforward pathways) with internal representations (carried by feedback pathways). A multi-scale, multi-species approach is essential for understanding the site of integration, computational mechanism and functional role of this processing. To improve our knowledge we must rely on brain imaging with improved spatial and temporal resolution and paradigms which can measure internal processes in the human brain, and on the bridging of disciplines in order to characterize this processing at cellular and circuit levels. We highlight apical amplification as one potential mechanism for integrating feedforward and feedback inputs within pyramidal neurons in the rodent brain. We reflect on the challenges and progress in applying this model neuronal process to the study of human cognition. We conclude that cortical-layer specific measures in humans will be an essential contribution for better understanding the landscape of information in cortical feedback, helping to bridge the explanatory gap.

## Highlights

• Understanding how the cortex integrates feedback and feedforward signals is central to understanding brain function.
• The data-driven framework of apical amplification which is hypothesized to have a central role in cognition is highlighted.
• Human neuroimaging data provides evidence for layer-specific cortical feedback relevant for theories of predictive feedback.

## 1 The layered cortex

**Fig. 1:**
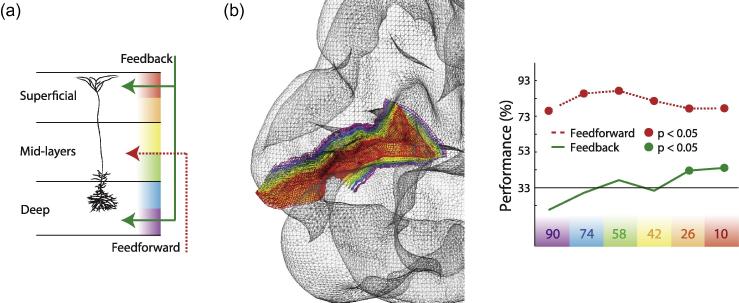
Bridging the gap between micro- and macroscopic properties of feedback in cortex. **a.** Feedforward and feedback pathways are found in distinct layers of cortex, with feedback terminating largely in superficial and deep layers (green arrows) and feedforward in mid-layers (red dashed arrow). An example layer 5 pyramidal cell is shown, as this is a prominent target cell type of cortical feedback. Feedback arrives to the apical dendrites of pyramidal neurons in L1 (and to interneurons), whereas feedforward input arrives to the somatic region. Pyramidal neurons thus have two integration sites; one at the top of the apical trunk and one at the soma (see Larkum for detail, 2013). Vertical colour bar depicts equidistant cortical depth sampling levels as has been studied with high-resolution brain imaging of early visual cortex (Muckli et al., 2015, see b). Depth sampling represents coarse approximations of layers and may not map directly onto anatomical layers. **b. Left:** Cortical reconstruction of the left hemisphere of a human subject (Muckli et al., 2015). Grid depicts cortical depth layers from superficial (red) to deep (purple). **Right:** Cortical depth-specific information decoding during feedforward and feedback visual processing for a representative human subject (using a support vector machine classifier), reproduced with permission from Muckli et al. (2015).

The integration of feedforward and feedback signals is important for healthy cognition and consciousness. In certain mental disorders, the cortex is deficient in integrating sensory signals with internal representations; during hallucinations, the brain fails to determine the (mis)match between its internal representation and the information it receives from the sensory environment, resulting in a conscious percept of a non-existent sound or sight for example (e.g. Horga, Schatz, Abi-Dargham, & Peterson, 2014). One endeavor of modern science is to understand human brain function in health and disease, for which we are required to enlist animal models for cellular and circuit level descriptions. Central to this effort is to understand processing in the neocortex, an area which makes up to 80% of the brain’s mass (Geschwind & Rakic, 2013). Given that feedforward and feedback inputs originate and terminate in different cortical layers (Fig. 1a, see Markov & Kennedy, 2013), it is advantageous to achieve the spatial scale to separate approximate representations of layers in human neuroimaging experiments. This field is emerging, with layer-resolved EEG, MEG and fMRI experiments gradually becoming more standard, though largely still in the healthy population. Alongside these measurement tools, we need paradigms in which we can access internal (i.e. non-sensory) signals (Chong, Familiar, & Shim, 2015, see also Petro & Muckli, 2016). Such paradigms are essential for mapping function to physiological measures, because feedforward and feedback processing have markedly different effects on (population) receptive fields. The role of feedforward processing is in signaling and transforming sensory inputs. In contrast, feedback processing is central to the enticing narrative that the brain predicts its environment (Clark, 2013, Park and Friston, 2013). Predictive processing may be important for guiding cognition and behaviour, and may be the core computation of the cortex upon which reward, attention, expectation and emotion act as modulators. Such network systems are central to the question of what is transmitted by top-down signals (Petro, Vizioli, & Muckli, 2014), in addition to sensory-specific feedback signals of complex features. We are able to coarsely approximate what information is contained in feedback signals in humans, for example, predictions about high level features of natural scenes (Morgan, Petro, & Muckli, 2016) and gratings (Chong et al., 2015). There are other important features of feedforward-feedback integration that are accessible to primate experimentation. For example, we need to understand how proximal to the sensory receptors that feedback exerts its effects; e.g. in vision, higher level processing acts on the primate lateral geniculate nucleus (Jones et al., 2015). Contributing to the question of when feedforward inputs are integrated with feedback, fluctuations in cortical ongoing activity that modulate perception reveal that internal modeling of forthcoming sensory inputs may precede their arrival to cortex (Hesselmann, Kell, & Kleinschmidt, 2008). We also know that feedforward and feedback signals act on different glutamate receptors (Self, Kooijmans, Supèr, Lamme, & Roelfsema, 2012) and are characterized by separate oscillatory rhythms (Bastos et al., 2015, van Kerkoerle et al., 2014). Top-down processing is observed in the alpha or beta range with feedforward processing carried by gamma and theta frequencies, suggesting that bottom-up and top-down processing serve different roles in communication and paving the way for the investigation into how feedback rhythms influence feedforward responses to sensory stimulation. Modeling work shows, for example, top-down beta rhythms can be important for gain control in superficial layers during stimulus processing via a process of inhibition (Lee, Whittington, & Kopell, 2013). Separating messages into different frequency bands might be a strategy to help keep message passing independent when needed. Similar to the multiplexing of frequencies in the radio, the sender and receiver can in principle tune into feedback and feedforward signals independently. The advantage for the brain is that depending on behavioural demands it might be necessary to give more weight to perceptual input or internal models. Despite these studies, the neuronal implementation of feedforward-feedback integration in cognition remains not fully conceptualized.

## 2 Apical amplification – two-compartment model of rodent pyramidal neurons

The principle targets of feedback in cortex are the distal tuft dendrites of layer 5 pyramidal neurons. These distal tuft dendrites extend up to layer 1 where 90% of inputs are from long-range feedback (Douglas & Martin, 2007). In a recent opinion paper, Matthew Larkum outlined how the cortex could achieve associative processing by the segregated arrival of feedback and feedforward inputs to distinct regions of a pyramidal neuron: the tuft and basal dendrites respectively (Larkum, 2013). A second action potential initiation zone (aside from that near the soma) can be found near the tuft of these deep layer 5 neurons. Here, feedback inputs arrive to these tuft dendrites and trigger Ca^2+^ spikes meaning that feedback inputs may have a greater role in determining the firing of pyramidal neurons than previously understood, because these Ca^2+^ spikes can convert a single somatic output spike into a 10 ms burst containing 2–4 spikes. As Larkum (2013) puts it; “counter-intuitively, far from being a minor influence on pyramidal cell firing, distal feedback input to the tuft dendrite could potentially dominate the input/output function of the cell”. With the coincident arrival of feedforward input to the somatic region, a back-propagated Na^+^ action potential generated in the axon facilitates the reaching of the threshold for dendritic Ca^2+^ spikes. Whilst it is appealing to conceive that such a process might also occur in human cortex, we need more evidence of bursting in monkeys or human tissue. Two photon calcium imaging is still most routinely used in awake rodents but some studies have used this technique in anaesthetized macaques, offering the potential to study dendritic signals in superficial layers of awake macaques in the future (Nauhaus, Nielsen, Disney, & Callaway, 2012). A candidate paradigm for this experiment would be figure-ground segmentation, which is known to include feedback influences to layers 1, 2 and 5 (Self, van Kerkoerle, Super, & Roelfsema, 2013).

The empirical data of Larkum and others are exciting given how we conceptualize and investigate the integration of sensory input with internal signals in the cortex. For example, this ‘double-integration site’ hypothesis of a pyramidal neuron could be incorporated into a neural network model. It would be of great interest to learn if it would facilitate performance in, for example, visual recognition. It is also important to understand how apical amplification (see Phillips, 2017) works when the brain represents internal models in the absence of feedforward input, which we know humans can do during working memory (Harrison & Tong, 2009). In this case, there would be no input to the somatic integration site, so there is nothing to be amplified yet we know a sensory representation is maintained in the system. A recent neural network model suggests that reward can strengthen the synapses which represent an attended stimulus using memory traces of useful, rewarding or predictable inputs. Moreover, this may work for stimuli not currently presented (Rombouts, Bohte, & Roelfsema, 2015). How apical amplification relates to computational theories of predictive feedback remains to be seen, but there are several intriguing possibilities. For example, if the feedforward input matches the contextual inputs or internal predictions sent to the tuft dendrites through feedback, the output may be amplified. In Adaptive Resonance Theory (ART), feedback provides hypotheses for object representations in the sensory signal. If the internal model is a good match for the sensory input, responses are enhanced (Grossberg, 2013). Coherent infomax claims that the cortex can amplify relevant inputs and suppress the irrelevant inputs (Phillips, 2017), and apical amplification provides a plausible physiological mechanism for this. In contrast, in hierarchical predictive coding, predictions of sensory input fed down the hierarchy serve to silence the predicted input, with a higher response only being seen in the case of a mismatch between prediction and sensory input (Friston, 2010). Theoretical similarities and discrepancies between predictive coding and apical amplification are described in detail (among other theories of cortical prediction) by Phillips (2017).

## 3 Feedback to superficial layers in human cortex

Rodent data revealing dissociable integration sites for feedback and feedforward inputs to pyramidal neurons is growing, and presents a compelling story of a generalized cortical processing mechanism (Larkum, 2013). The majority of the human brain is composed of cortex, and the majority of cortex is pyramidal neurons (Nieuwenhuys, 1994). If a similar mechanism exists in humans, how conceivable is it to empirically study putative feedforward (basal) and feedback (tuft) activity in pyramidal neurons in the human brain? Such investigation would at least require layer-specific resolution, but is it realistic to exploit the improving resolution of state-of-the-art noninvasive techniques, employing cognitive paradigms, to study the organizational principles of cortex revealed by microscopic recordings (such as dendritic patch clamping and calcium imaging)? High-field, high-resolution functional magnetic resonance imaging (fMRI) provides sub-millimeter measures of human cortex at the level of layers (Olman et al., 2012) and columns (Yacoub, Harel, & Ugurbil, 2008). We recently revealed the information patterns carried in cortical feedback to layers of human visual cortex using high field (7T) fMRI (Fig. 1b, Muckli et al., 2015). We retinotopically mapped the region of V1 responding to a masked portion of a complex natural scene visual stimulus. We then delineated the cortical representation of the masked region of cortex into coarse representations of laminae. We took functional data specific to each of these six depth layers and using multivariate pattern classifiers were able to readout information that was predictive of the scene behind the mask. Interestingly, we only found this contextual feedback in the superficial layers, where feedback inputs arrive to the tuft dendrites of pyramidal neurons. Furthermore, fMRI reflects neuronal energy consumption which includes dendritic activity (Logothetis, 2008, Muckli, 2010). The profile of BOLD in cortical depths was not predictive of the decoding patterns in the same depths; moreover, we replicated the finding using a GRASE sequence which has improved specificity compared to GE-EPI (De Martino et al., 2013). This is important because deoxygenated blood filters in a manner perpendicular to cortical layers, back towards the pial surface where there are large draining veins (Blinder et al., 2013). This data prompt many questions, not least what is the precise information contained in the feedback signal and, if a process similar to apical amplification is present in our data, what can we expect of the action potential output of a pyramidal neuron when scene feedback can only amplify (or disamplify) the sparse feedforward signals representing a masked region?

We acknowledge the disparity of spatial and temporal dynamics between BOLD signals and dendritic and somatic spikes. As mentioned above, the superficial layer specificity of feedback signals observed in humans is a potential correspondence to apical amplification, such that in apical amplification feedback arrives to the tuft dendrites in outer layers. However there is an obvious limit as to which BOLD signals are informative about cellular processes in apical amplification, if apical amplification exists in humans. Other promising approaches include layer-resolved electrophysiological recordings (Halgren et al., 2015) and the modeling of laminar-specific MEG signals (Troebinger, López, Lutti, Bestmann, & Barnes, 2014). Such data will contribute decisive information about the temporal dynamics of feedforward and feedback processing in human cortex. However none such human data will achieve individual neuron resolution, which is at the crux of apical amplification. Calcium imaging of cortical layers in awake monkeys is not yet routine due to technical limitations. However, hopefully in future this type of data will provide the necessary resolution to study microcircuits during tasks such as working memory, figure ground segmentation and visual occlusion.

## 4 Conclusion

Layer-specific brain imaging in humans constitutes a shift in the spatial scale at which we can investigate feedback processing. Narrowing the gap from microscopic to macroscopic signals is gaining momentum with advancing technology and collaborations spanning multi-scale and multi-species data. It will help to have common paradigms and common conceptual frameworks. There are inescapable caveats such as the relevance of human cognitive paradigms to rodents and monkeys, and inherent differences in cortex (for example mice do not have a foveal representation, and in general have poor vision). That said, if we take vision as an example, rodents perform well in perceptual and behavioural tasks, and there are overlaps in anatomy and receptive field properties between rodents, monkeys and humans. Apical amplification is a candidate for a common conceptual framework for which we can take a multi-scale (inherently multi-species) approach to understanding the role of cortical feedback in how neurons perform inference. This rodent data will be bolstered by primate data in future, and such data will undoubtedly inform neural network models. With regards to humans, evidence for the role of input to the apical tufts of pyramidal cells in cognition through amplification of their responses to feedforward input is providing new insights (Phillips, 2017). We can learn about the properties of feedback when feedforward input is removed (Muckli & Petro, 2013), for example, that it is predictive or constructive (Chong et al., 2015). Moreover, the investigation of healthy human cortex at the level of layers will facilitate better understanding of the role of feedback in cognition. The layer-resolution offered by sub-millimeter fMRI holds immense potential for studying brain disorder, as counter-stream processing is a key target area for understanding schizophrenia (e.g. Horga et al., 2014), autism and attention deficit hyperactivity disorder (Gonzalez-Gadea et al., 2015), and depression (Chekroud, 2015). To name just a few of the remaining open questions: If apical amplification has a central role in human cognition, does it vary with changes in pyramidal cell morphology throughout the cortex? What other mechanisms could be responsible for the integration of feedforward and feedback signals, and how might that process be incorporated in a functional large-scale circuit? Until we understand how the brain’s internal and external worlds are combined, we are missing an essential puzzle piece in our understanding of constructive brain processes.
